# The growing evidence for mental health rehabilitation services and directions for future research

**DOI:** 10.3389/fpsyt.2023.1303073

**Published:** 2023-11-20

**Authors:** Helen Killaspy, Christian Dalton-Locke

**Affiliations:** ^1^Division of Psychiatry, University College London, London, United Kingdom; ^2^Camden and Islington NHS Foundation Trust, London, United Kingdom

**Keywords:** mental health, rehabilitation, healthcare records, current evidence, future research

## 1 Introduction

Mental health rehabilitation services work with people with particularly severe mental health problems to enable them to gain the skills and confidence for successful community living. The majority of people requiring these specialist services have a primary diagnosis of a psychotic disorder, such as schizophrenia, schizoaffective disorder or bipolar affective disorder that has not responded adequately to usual treatments. Often, people will have ongoing symptoms such as hallucinations and delusions that may cause them ongoing distress, alongside so called “negative” symptoms that affect their motivation and organizational skills. Additional physical and mental health comorbidities often complicate recovery further. Together, these problems make it difficult for the person to engage in day-to-day activities (such as self-care, housework, shopping, cooking, and budgeting) and they often struggle with interpersonal skills and become increasingly isolated and marginalized from community life. Around 20–25% of people newly diagnosed with psychosis will go on to develop these kinds of issues (1–3). Increasingly, they are described as experiencing “complex psychosis”. Due to their many difficulties, they often require recurrent and lengthy periods of inpatient treatment and, in the UK, it has been estimated that they account for around half the total spend on mental health (4).

Mental health rehabilitation services are staffed by multidisciplinary teams including psychiatrists, psychologists, nurses, social workers, and occupational therapists. They provide a range of interventions, including:

- Optimisation of psychotropic medication, often involving complex regimes including clozapine, augmentation strategies, and the management of side effects.
- Assessment, monitoring, and management of co-morbid physical health problems and liaison with medical colleagues in primary and secondary care.
- Psychological assessment, formulation, and psychological interventions including individual talking therapies, group therapy, family work, and psychometric testing.
- Occupational therapy interventions to enhance confidence and competence with activities of daily living.
- Enabling group activities (social, leisure, or occupational) to improve interpersonal skills; these should be offered daily in inpatient rehabilitation services and at least weekly in community settings.
- As people progress in their recovery, they are supported to engage in leisure, educational, and vocational opportunities in the community (e.g., gym, cinema, cafes, Recovery College courses, adult education, supported employment, and voluntary work). Therefore, developing partnerships with local voluntary organizations, colleges, supported employment services, and leisure and entertainment venues, is another key task for rehabilitation services.
- Providing support to families and carers (in addition to any specific family therapy).

Therapeutic optimism is critical in mental health rehabilitation. To provide support effectively, it is vital that staff are appropriately supported themselves through supervision and reflective practice sessions where challenges can be shared and addressed, including acknowledgment and management of any feelings of pessimism about people's potential for recovery.

Historically, rehabilitation services have been under-represented in research. Fortunately, this situation has started to change and there is now a growing evidence base demonstrating the effectiveness of mental health rehabilitation services for people with complex psychosis.

## 2 Evidence for mental health rehabilitation and its limitations

Large-scale national research programmes in England have shown that around two-thirds of people who are admitted to an inpatient mental health rehabilitation unit achieve successful discharge from hospital within a year, without subsequent readmission or community placement breakdown (5). Furthermore, over 40% continue to progress in their recovery once in the community, graduating successfully from higher levels of supported accommodation to more independent settings within three years (6). One, small, longitudinal study in North London followed people who had used local rehabilitation services over a period of five years and found that two-thirds moved successfully to more independent settings, but, of note, only 10% were able to live independently, highlighting the high level of disability and ongoing support needs of people with complex psychosis (7). These national studies have also identified that mental health rehabilitation services that adopt more of a “recovery” orientated approach (summarized as an individualized, collaborative way of working that emphasizes hope and supports the person to identify and work toward their chosen goals) are more effective in terms of enabling successful hospital discharge and greater independence in the community (5, 6). Researchers in the Netherlands have developed a model to improve recovery-based practice in mental health rehabilitation services (8), and an associated model fidelity tool (9). Evaluation by the same team has recently been completed and the associated publication is keenly anticipated.

A number of “before and after” studies conducted in the UK (10), North America (11), and Australia (12) have shown that acute inpatient service use is reduced when people with complex psychosis have access to mental health rehabilitation services and this is associated with reduced costs of care (10). These results provide encouraging evidence that when people have access to mental health rehabilitation services, there is good reason to be optimistic about their future and investment in these services is worthwhile. However, there are limitations to these studies including small samples sizes and relatively short before and after periods. Also, the lack of a comparable control group means that “regression to the mean” (naturalistic clinical improvement over time) may explain at least some of the findings.

The “gold standard” study design that overcomes these issues is the randomized controlled trial (RCT). However, when services, such as mental health rehabilitation services, are already well established, randomization to a comparison intervention can be challenging or even potentially unethical. For example, a feasibility trial to compare two types of supported housing in England screened 1,432 people but only eight were randomized. Barriers included concerns about accommodation being decided at random and a perceived lack of equipoise among clinicians who felt that individuals needed to follow the usual graduated care pathway and “step down” from higher to lower levels of supported housing (13).

## 3 The potential utility of electronic healthcare records in research

The increasing use of healthcare records in research may help to address some of the limitations and logistical obstacles encountered in previous studies. The Clinical Records Interactive Search (CRIS) deidentifies and structures electronic healthcare records making the data available to researchers (14–16). CRIS has been successfully deployed in several NHS mental health Trusts across England, but it is most well established in the organization where it was developed, the South London and Maudsley NHS Foundation Trust (SLaM). This Trust provides inpatient and community mental health care to residents of the London boroughs of Lambeth, Southwark, Lewisham and Croydon, a combined population of around 1.2 million people. As of January 2020, the CRIS database for SLaM contained records pertaining to more than 340,000 adults, spanning from 2007/2008 when the Trust switched from paper to electronic records (17).

Over the last 15 years, CRIS has been used in hundreds of peer-reviewed studies on a range of topics, including a comparison of inpatient service use before and after admission to the National Psychosis Unit (18). The National Psychosis Unit is similar to an inpatient rehabilitation service in providing specialist treatment to people with complex psychosis, however, the service operates at a national rather than local NHS Trust level. The study used CRIS data linked to Hospital Episodes Statistics, a national database containing information about all NHS hospital admissions, for mental health or physical health problems, across England. This data linkage was critical to the study given that the local CRIS database only contains data on mental health hospital admissions within SLaM, but the National Psychosis Unit receives patients from across the country.

With access to this wealth of data, the researchers were able to compare inpatient service use two years before and two years after an admission to the National Psychosis Unit for 147 individuals (18). They found that inpatient service use reduced from a mean of 335 days (SD 273) before the National Psychosis Unit admission to 199 days (SD 262) afterwards. Although this study had a smaller sample size than one of the aforementioned before and after studies (12), it provides greater balance in terms of the sample size and the length of the before and after periods. Furthermore, as this area of research continues to develop, both in terms of the amount of healthcare records available and linkage with other databases, the sample sizes, duration of records, and potential uses (e.g., investigating outcomes other than inpatient service use), will increase. It is also worth noting that there are many other systems like CRIS being used for similar research internationally. For example, the Nordic registers have been described as a “potential goldmine for clinical research” (19). Nevertheless, the CRIS National Psychosis Unit study has the same issue as the other before and after studies when it comes to the limited extent to which causality can be inferred and regression to the mean.

There is an ongoing debate over the extent to which causality can be inferred from the analysis of observational datasets, such as those derived from healthcare records (20). Wang et al. (21) have shown that it is possible to emulate an RCT using healthcare record datasets, but the viability of this depends on the specific research question, study design, and the data available in the observational dataset. Wang et al.'s study (21) emulated 32 randomized controlled trials using US health insurance datasets, but all were assessing the effectiveness of medications for various medical conditions. Whether it is possible to extrapolate these techniques and infer causality from observational datasets when the intervention is as complex as mental health rehabilitation remains unexplored.

## 4 Current evidence and clinical guidance

Whilst healthcare records may provide a potentially fruitful avenue for research into mental health rehabilitation services in the future, studies over the last decade or so have culminated in the publication of the first Clinical Guideline on mental health rehabilitation by the National Institute of Health and Care Excellence in August 2020 (22). The Guideline provides evidence-based recommendations on the specific treatments and support that mental health rehabilitation services should provide. It describes rehabilitation services as including inpatient and community based rehabilitation units, and community mental health rehabilitation teams that provide specialist clinical input to people living in highly supported accommodation. These services are organized into a local rehabilitation care pathway that is embedded in the wider local mental healthcare system. The Guideline emphasizes that all rehabilitation services should adopt a recovery orientated approach, which can be summarized as a personalized and collaborative way of working, that enables the person with complex psychosis to identify specific goals and tailors support to help them work toward them. This recommendation was based on evidence from the two large cohort studies in England mentioned previously (5, 6). Despite the absence of a control group, cohort studies are appropriately designed to identify the characteristics of services that assist people with their recovery process, and they can also help to identify the characteristics of people who may benefit more (and less) from rehabilitation services. For example, the national cohort study of inpatient rehabilitation services in England identified that people who had been in hospital longer and those with a history of fire-setting were less likely to achieve successful community discharge, while those with better social skills and greater engagement in activities were more likely to do so (5). Similarly, White et al. (23) found that people who engaged more with their treatment (including medication, occupational therapy, and addressing substance misuse) during admission were less likely to be readmitted.

These studies signal the importance of ensuring that staff are supported to offer individualized approaches that encourage and enable people to engage in the specific biopsychosocial interventions that can help them. This is particularly important in rehabilitation services where the very nature of people's complex mental health problems can cause apathy and therapeutic pessimism in the clinical team. The NICE Guideline specifically recommends targeted support for clinicians, such as reflective practice groups and individual supervision, to address this (22).

## 5 Recommendations for future research

Despite significant advances, many research questions remain in this field and the NICE committee therefore made a number of recommendations for future research where they identified that evidence was particularly scant (22). These included investigation of: the effectiveness of rehabilitation services for people at an earlier stage of psychosis; the role of peer support in rehabilitation services; group interventions for improving social skills for people with complex psychosis; the effectiveness of highly specialist rehabilitation services; and the role of the independent sector. A national study in England addressing the last of these is currently underway (the ACER study: Assessing the Clinical and cost-Effectiveness of inpatient mental health Rehabilitation services provided by the NHS and independent sector). In conclusion, although this is a growing field, there is still plenty of work for researchers who want to understand how to help people with complex psychosis to optimize their recovery.

## Author contributions

HK: Writing—original draft, Writing—review & editing. CD-L: Writing—original draft, Writing—review & editing.
